# Pros and Cons of Inpatient SGLT2i Use for Hyperglycemia and Heart Failure

**DOI:** 10.1210/jendso/bvae229

**Published:** 2025-01-15

**Authors:** Hayley Fried, Yael Tobi Harris, Rifka Schulman-Rosenbaum

**Affiliations:** Donald and Barbara Zucker School of Medicine at Hofstra/Northwell, Department of Medicine, Lenox Hill Hospital, Northwell Health, New York, NY 10075, USA; Donald and Barbara Zucker School of Medicine at Hofstra/Northwell, Division of Endocrinology, Diabetes and Metabolism, Long Island Jewish Medical Center, Northwell Health, New Hyde Park, NY 11040, USA; Donald and Barbara Zucker School of Medicine at Hofstra/Northwell, Division of Endocrinology, Diabetes and Metabolism, North Shore University Hospital, Northwell Health, Manhasset, NY 11030, USA; Donald and Barbara Zucker School of Medicine at Hofstra/Northwell, Division of Endocrinology, Diabetes and Metabolism, Long Island Jewish Medical Center, Northwell Health, New Hyde Park, NY 11040, USA

**Keywords:** sodium-glucose cotransporter 2 inhibitors, inpatient type 2 diabetes mellitus management, inpatient hyperglycemia management, inpatient heart failure management, inpatient clinical guidelines, sodium-glucose cotransporter 2 inhibitor contraindications

## Abstract

Sodium-glucose cotransporter 2 inhibitors (SGLT2is), originally approved by the US Food and Drug Administration for glycemic control in type 2 diabetes mellitus (DM2), have shown substantial cardiovascular and renal benefits, leading to their expanded use in managing heart failure (HF) and chronic kidney disease in the outpatient setting. Despite these benefits, their use for inpatient hyperglycemia management is not universally endorsed due to safety concerns and inadequate data. However, emerging evidence suggests potential advantages of initiating SGLT2i treatment for patients during hospitalization in the setting of HF. While SGLT2is are not recommended for managing inpatient hyperglycemia, initiation during hospitalization for HF provides significant benefits. We review the current literature on the pros and cons of using SGLT2is in hospitalized DM2 and HF patients and provide guidance on careful patient selection and risk mitigation for inpatient use.

Although sodium-glucose cotransporter 2 inhibitors (SGLT2is) were initially developed and approved by the US Food and Drug Administration (FDA) for the glycemic management of type 2 diabetes mellitus (DM2), their effect on cardiac and renal health has led to an expanded use in clinical practice. In addition to improving hemoglobin A_1c_ with minimal risk of hypoglycemia, SGLT2is can significantly reduce the risk of cardiovascular hospitalization and mortality in individuals with heart failure (HF) and enhance diuresis in patients with acute HF [1-6].

The 2024 American Diabetes Association Standards of Care recommend SGLT2is for outpatient management of patients with DM2 with established or high risk of atherosclerotic cardiovascular disease, HF, or chronic kidney disease, irrespective of glycemic control. They also advise continuing SGLT2is in hospitalized patients with DM2 and HF after acute illness recovery, if no contraindications exist [7]. Additionally, the 2022 joint guideline from the American College of Cardiology, the American Heart Association, and the Heart Failure Society of America recommend SGLT2is for treatment of HF with reduced ejection fraction and mildly reduced or preserved ejection fraction, with continuation or initiation while hospitalized and prior to discharge, if no contraindications exist [8].

The increasing recognition of their benefits has generated curiosity about SGLT2i use in inpatient settings; however, research on their inpatient safety and efficacy has not kept pace with their established widespread applications in outpatient care. Here we explore the rationale for use of SGLT2is in inpatient settings both for hyperglycemia and HF indications, and we discuss precautions required with their use.

## Glycemic Control in Hospitalized Patients

Inpatient hyperglycemia, defined as serum glucose concentration greater than or equal to 140 mg/dL [9], in patients with or without a prior diagnosis of diabetes, leads to a higher risk of complications, mortality, prolonged hospital stays, increased infections, intensive care unit admissions, and greater need for transitional care after discharge [10]. This has influenced clinical practice guidelines, leading to the broad adoption of an insulin basal-bolus regimen with a correctional scale as the standard of care for hyperglycemia in hospitalized patients [7, 9, 11]. However, insulin therapy increases hypoglycemia risk, which is associated with increased length of stay, morbidity, and mortality [12]. The development of newer DM2 pharmacotherapy agents, which offer different mechanisms of action with nonglycemic benefits and a lower risk of hypoglycemia, has prompted a reevaluation of the traditional basal-bolus approach to inpatient hyperglycemia management in noncritical care settings [13].

Few randomized controlled trials have compared insulin vs noninsulin treatments for managing hyperglycemia in hospitalized patients with DM2 and noncritical illnesses. Existing studies have shown that dipeptidyl peptidase-4 inhibitors (DPP4is) reduce insulin requirements and hypoglycemia events compared to insulin, and therefore may be appropriate for glycemic management of select patients with mild hyperglycemia hospitalized for noncritical conditions [9, 14]. Recent large-scale clinical trials have highlighted the benefits of SGLT2is in outpatient settings, leading to speculation about their potential utility in inpatient settings.

A recent Veterans Affairs cohort study on continuing SGLT2is from outpatient to inpatient settings in patients with DM2 found lower mortality rates, shorter hospital stays, and no increased risk of acute kidney injury (AKI). However, the study did not report glycemic control outcomes [15]. Despite suggesting potential benefits, the observational study has several limitations, such as selection bias, lack of randomization, and limited generalizability. Patients who continued SGLT2is were more likely to have cardiovascular disease and HF, which potentially influenced mortality rates. The study sample was primarily White male veterans, limiting its broader applicability. Without randomization, the observed associations may be confounded by illness severity, as patients with more severe conditions may have had SGLT2is discontinued, or clinical management differences. Therefore, while the study offers valuable insights, its findings should be interpreted cautiously. Current clinical guidelines do not recommend the use of SGLT2is for managing inpatient hyperglycemia due to inconclusive safety data and insufficient evidence regarding their effectiveness in the inpatient setting [7, 11, 16].

### Hypoglycemia

While agents that reduce inpatient hypoglycemia have value, a retrospective study comparing DPP4is and SGLT2is for inpatient hyperglycemia management showed no difference in glycemic control, length of stay, or mortality [17]. As a result, there is no clear advantage to using SGLT2is over DPP4is in the inpatient setting for mild hyperglycemia management, especially given the increased risk of adverse effects. These include concerns about reduced glycemic efficacy with worsening renal function, as well as the risks of diabetic ketoacidosis (DKA), euglycemic diabetic ketoacidosis (eDKA), genitourinary infections, and hypovolemia [16].

### Renal Function and Glycemic Efficacy

As renal function declines, glucose delivery to the kidneys is reduced, diminishing the glucosuric effect of SGLT2is. With lowering of the estimated glomerular filtration rate (eGFR), the antihyperglycemic effect of SGLT2is wanes and may become ineffective when eGFR is less than 30 mL/min/1.73 m^2^ [1, 18].

During hospitalization, the dynamic nature of illness can cause fluctuations in renal function, and patients with diabetes are particularly susceptible to AKI [19]. Factors such as infection, sepsis, hypovolemia, and exposure to nephrotoxic agents like contrast media and antibiotics, place hospitalized patients at an increased risk for worsening renal function [19]. The decline in the efficacy of SGLT2is in the setting of impaired renal function presents a challenge, as it may lead to hyperglycemia, undermining the therapeutic benefits of these agents.

While SGLT2is reduce progression of diabetic kidney disease in the outpatient setting, it is generally recommended to discontinue use in hospitalized patients at increased risk of AKI [7, 11]. Additionally, a lack of studies exists assessing the inpatient use of SGLT2is specifically for renal indication.

### Diabetic Ketoacidosis and Euglycemic Diabetic Ketoacidosis

DKA is a severe, life-threatening complication that can develop in individuals with diabetes requiring prompt recognition, diagnosis, and treatment for survival [20]. DKA is marked by hyperglycemia (blood glucose ≥200 mg/dL) or a history of diabetes, elevated ketones (β-hydroxybutyrate concentration ≥ 3.0 mmol/L or urine ketone strip 2+), and metabolic acidosis (pH <7.3 or bicarbonate concentration <18 mmol/L) [21]. eDKA occurs with ketosis and acidosis but blood glucose of less than 200 mg/dL [21]. DKA and eDKA have both been associated with SGLT2i use and can be triggered by risk factors such as fasting, low-carbohydrate diet, withholding of home insulin doses, surgery, infections, trauma, glucocorticoid use, cirrhosis, and alcohol use [20].

Although SGLT2is are linked to DKA and eDKA, risk levels vary between studies [22]. A recent meta-analysis of randomized controlled trials and cohort studies assessing SGLT2i use in hospitalized patients found a higher, though not statistically significant, incidence of ketoacidosis. These results may underestimate the true risk in real-world settings, where monitoring may be less stringent. Additionally, the majority of included patients did not have DM2, reducing the risk of ketoacidosis, and patients at high risk of DKA or clinical instability are often excluded from trials [23]. Inpatient settings introduce additional risks, such as infection, stress, surgery, volume status changes, and diet alteration or nothing-by-mouth, potentially increasing DKA risk compared to outpatient settings. The FDA advises discontinuing SGLT2is 3 to 4 days prior to procedures [24]. However, this approach is not always feasible, particularly in cases involving unanticipated urgent surgery. One retrospective study found that 1.1% of patients using SGLT2is and undergoing urgent surgery developed eDKA, compared to just 0.17% in those undergoing nonurgent surgery [25]. Another retrospective study highlighted this issue, finding that eDKA occurred in 15.4% of SGLT2i users after cardiac surgery [26].

A retrospective, multicenter cohort study investigating the incidence of DKA in hospitalized patients receiving SGLT2is for hyperglycemia found that 38% of these patients developed DKA, compared to just 2% in those not receiving SGLT2is, resulting in an odds ratio for DKA of 37.4 (*P* < .001), with most cases occurring in the context of surgery and prolonged fasting [27].

The Dapa-Hospital trial found that cardiac surgery patients on dapagliflozin with basal-bolus insulin had a higher incidence of severe ketonemia than those on insulin alone, but no DKA cases were reported [28]. This may be due to the trial's serum bicarbonate cutoff of 15 mmol/L, while current DKA guidelines use a threshold of bicarbonate below 18 mmol/L [21]. Additionally, the dapagliflozin group required similar insulin doses to maintain euglycemia, with the presence of insulin therapy likely preventing DKA [28]. Often patients’ insulin regimens are held on admission, or they receive fasting instructions pending procedures or surgery, which can raise the risk of DKA in SGLT2i users.

### Genitourinary Infections

Patients with DM2 are more prone to genitourinary infections due to chronic hyperglycemia, which increases glucose in the urine, promoting bacterial and fungal growth, and impaired immune function [29]. Conditions such as urinary retention, neuropathy, and poor circulation further increase the risk of urinary tract infections and genital mycotic infections [29]. The use of SGLT2is, which induces glucosuria, has been linked to a higher incidence of genitourinary infections, particularly in the outpatient setting, leading to medication discontinuation in older patients with DM2 [30, 31]. Although most infections are mild genital mycotic infections that respond to standard treatment, hospitalized patients with multiple risk factors, such as immunosuppression, AKI, catheterization, and immobility, are at risk for more severe infections, including sepsis and delayed recovery [30]. Despite these concerns, data on genitourinary infections in hospitalized patients using SGLT2is are scarce, highlighting the need for further research. Current guidelines recommend caution in using SGLT2is for hyperglycemia management in hospitalized patients, especially those at high infection risk [7, 11].

### Hypovolemia

Hypovolemia can cause weakness, syncope, and dizziness, which increases fall risk, a serious issue in older adults that leads to higher morbidity, mortality, extended hospital stays, and increased health care costs [32]. A meta-analysis of outpatient SGLT2i use found an increased risk of hypovolemia with SGLT2i use, especially in patients aged 65 years or older or with a baseline eGFR of less than 60 mL/min/1.73 m^2^ [33]. Given that hospitalized individuals have more precarious volume statuses, this risk is heightened, thus assessing for hypovolemia is crucial prior to initiating SGLT2is in the inpatient setting.

### Summary of Recommendations

The continuation or initiation of SGLT2is is not recommended for inpatient management of hyperglycemia due to several risks. These medications are associated with an increased risk of DKA, exacerbated by factors common in the hospital setting, such as fasting, infection, and surgery. SGLT2is promote glucosuria and ketone production, which can lead to DKA, particularly when combined with the stressors and volume changes that occur during hospitalization. Additionally, SGLT2is can increase the risk of genitourinary infections, which can lead to severe complications. The heightened risk of hypovolemia further complicates their use, as SGLT2is are known to promote diuresis and may worsen hypovolemic conditions. While there may be a subset of patients for whom SGLT2is are safe in the inpatient setting, because the overall risk for many patients is high and there are no inpatient data showing improved glucose control, our perspective is to err on the side of holding. Given the lack of inpatient trials specifically focusing on SGLT2is for hyperglycemia management and given ample data supporting the safe use of basal-bolus insulin or DPP4is, current guidelines advise against using SGLT2is for managing hyperglycemia in hospital settings [7, 9, 11]. This stance may evolve as new data emerge in this area.

## Heart Failure

Although SGLT2is may not be ideal for managing inpatient hyperglycemia, studies highlight their cardiovascular benefits of starting treatment during hospitalization. This has shifted their role to essential components of guideline-directed medical therapy (GDMT) for HF and potential use in hospital settings.

HF results from structural or functional cardiac issues, marked by elevated natriuretic peptide levels or evidence of congestion [34]. DM2 significantly increases HF risk, with 20% to 40% of HF patients having DM2 and a 30% higher risk of HF hospitalization compared to those without DM2 [35].

### Sodium-Glucose Cotransporter 2 Inhibitor Use in the Outpatient Setting

To meet FDA cardiovascular safety requirements, extensive trials over the past decade have demonstrated that SGLT2is reduce the risk of cardiovascular hospitalization and mortality in individuals with HF, both with preserved and reduced ejection fraction, regardless of DM2 status, yielding protective cardiac outcomes. This has expanded their use beyond the original purpose. Key clinical trials that have influenced the inclusion in GDMT for HF management, as endorsed by the major cardiology organizations, include EMPA-REG OUTCOME [1], CANVAS AND CANVAS-R [2], DECLARE-TIMI 58 [3], DAPA-HF [4], EMPEROR-REDUCED [5], and EMPEROR-PRESERVED [6]. Although these trials were primarily conducted in outpatient settings, secondary analyses revealed a notable pattern in the time to clinical benefit. These studies demonstrated that SGLT2is provide early and sustained clinical advantages in HF. For example, empagliflozin showed significant reductions in cardiovascular death and hospitalizations within just 12 days of randomization, prompting interest in starting SGLT2is before hospital discharge [5].

### Sodium-Glucose Cotransporter 2 Inhibitor Use in the Inpatient Setting

The SOLOIST-WHF [36], EMPULSE [37], DICTATE-AHF [38], and EMPA-RESPONSE-AHF [39] trials investigated the initiation of SGLT2is in hospitalized patients with acute decompensated HF. Despite ending termination due to funding issues, the SOLOIST-WHF trial showed reduced cardiovascular death, HF hospitalizations, and urgent HF visits in patients with DM2 [36]. The EMPULSE trial found significant clinical benefits from initiating empagliflozin during hospitalization, including improvements in a composite of death from any cause, fewer HF events, increased time to first HF event, and a decrease in a 5-point or greater difference in change from baseline in the Kansas City Cardiomyopathy Questionnaire Total Symptom Score, after 90 days of therapy, regardless of ejection fraction, HF type, or DM2 status [37]. The DICTATE-AHF trial demonstrated that dapagliflozin enhanced diuresis and natriuresis, resulting in shorter hospital stays [38]. Additionally, the EMPA-RESPONSE-AHF trial reported that initiating empagliflozin during admission reduced the composite end point of worsening HF, rehospitalization, or death at 60 days, irrespective of DM2 status [39]. Despite these findings, SGLT2is are underused in hospitalized HF patients, partly due to concerns about adverse effects and insufficient research. Delaying GDMT is linked to never starting therapy [40]. Therefore, strategies to promote early SGLT2i initiation during hospitalization or at the time of discharge are crucial.

Initiating SGLT2i therapy during hospitalization has several benefits: It promotes rapid clinical improvements, optimizes medication regimens, allows for close monitoring of disease and drug tolerance, facilitates assessment of insurance coverage, and enables patient education, all of which address clinical inertia [41].

Hospital settings provide an opportunity for close monitoring of adverse effects and immediate assessment of laboratory values, which facilitates expedited adjustment of medications, including antihyperglycemic agents, antihypertensives, and diuretics. Additionally, hospitalization offers an opportunity for patient education, management, and adherence [42]. Given the high cost and often complex prior authorizations associated with these drugs, hospital admission allows for the identification of patients who might need assistance with payment through cost-assistance programs.

### Summary of Recommendation

SGLT2is are indicated for use in hospitalized patients with HF; careful patient selection, as well as close monitoring and titration, are crucial to mitigate adverse effects. While the risk of ketoacidosis is increased in patients with DM2 using SGLT2is, there are case reports of SGLT2i-induced ketoacidosis occurring in patients without diabetes [43]. To further reduce risk of adverse events, SGLT2i initiation should be delayed until closer to discharge or at the time of discharge, ensuring the patient's clinical stability prior to medication initiation.

## Conclusion

Although originally developed for glycemic control, SGLT2is have proven benefits for cardiovascular health, leading to their expanded use in HF management. For most hospitalized patients with DM2 and without HF, basal-bolus insulin, or in the case of mild hyperglycemia, DPP4i, remains the standard of care. Although not ideal for managing inpatient hyperglycemia, initiating SGLT2is during hospitalization for HF can offer benefits and address factors contributing to clinical inertia. The most recent cardiology and endocrinology societies’ recommendations include starting or continuing SGLT2is for hospitalized patients with HF with or without DM2, after resolution of acute illness, provided there are no contraindications [7, 8, 11].

**Figure 1.**
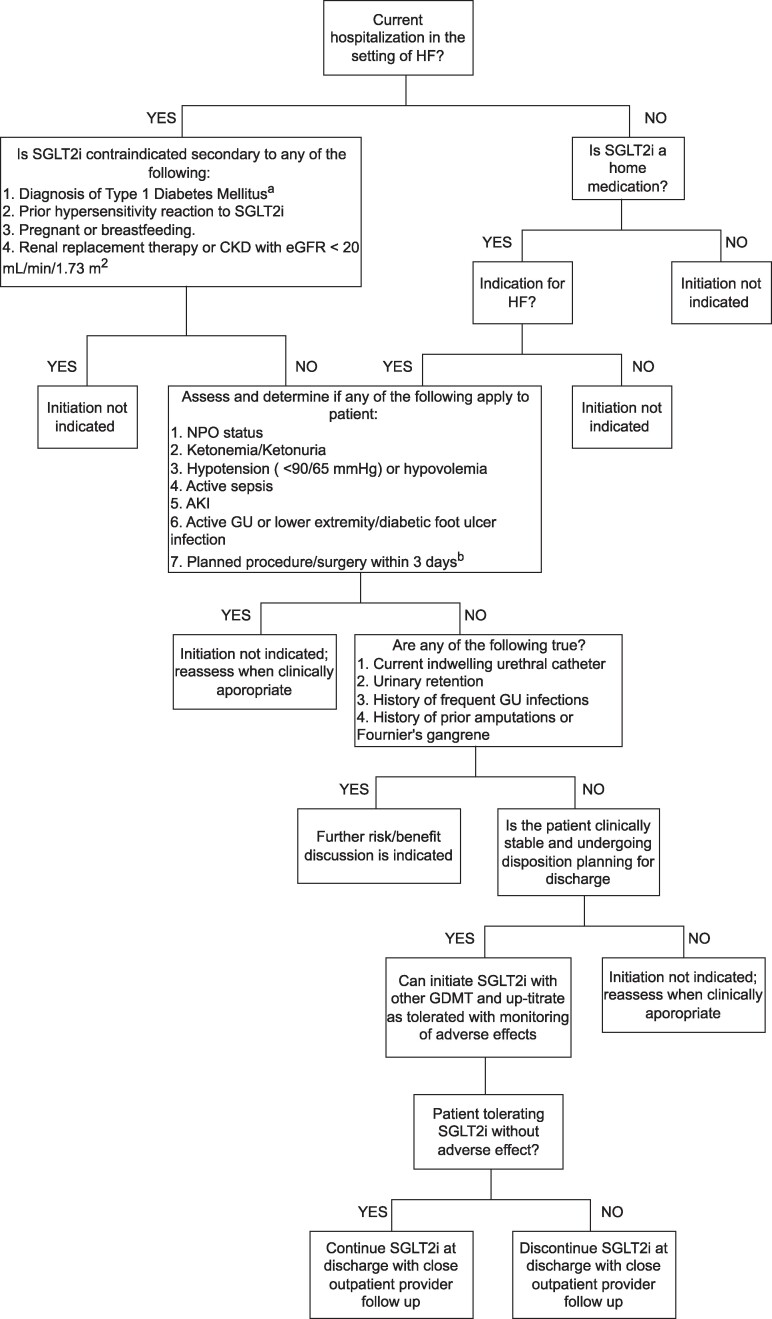
Proposed algorithm for the initiation of SGLT2is in the inpatient setting for HF. AKI, acute kidney injury; CKD, chronic kidney disease; eGFR, estimated glomerular filtration rate; GDMT, guideline-directed medical therapy; GU, genitourinary; HF, heart failure; NPO, nil per os; SGLT2i, sodium- glucose cotransporter 2 inhibitor. ^a^SGLT2is may be used off-label in type 1 diabetes mellitus, but are not recommended for inpatient use in this population. ^b^Ertugliflozin should be held 4 days prior to planned procedure/surgery.

When considering SGLT2is for inpatient HF treatment, meticulous patient selection is critical (Fig. 1). It is essential to understand contraindications to use and when to avoid in high-risk scenarios. To maintain safety standards, hospital-wide education for health-care providers and pharmacy staff is necessary. Implementing SGLT2is should be delayed until the patient is clinically stable and near discharge to minimize adverse effects and reduce clinical inertia. During the initiation of SGLT2i therapy, continuous monitoring of vital signs, frequent laboratory tests to assess euglycemia, ketosis, and renal function, and, if necessary, urinalysis and complete blood counts to detect infections, are crucial. Integrating safety parameters and checks into the ordering system can help ensure appropriate patient selection and minimize potential harm. Hospital protocols should be instituted to guide inpatient SGLT2i ordering as well as how to handle urgent procedures that may arise without adequate hold time for patients requiring the nothing-by-mouth state [44].

Given the limited data on the use of SGLT2is in inpatient settings, additional research is needed to comprehensively assess their associated risks and benefits. Future studies should focus on generating more data regarding the use of SGLT2is for inpatient glycemic control, developing protocols that incorporate concurrent noninsulin therapies, evaluating patient outcomes beyond glycemic control, and integrating patient perspectives and preferences to facilitate shared decision-making between patients and providers. Randomized controlled trials could provide valuable insight into determining the optimal timing for initiating SGLT2is prior to discharge.

## Data Availability

Data sharing is not applicable to this article as no data sets were generated or analyzed during this study.

## Abbreviations

AKI: acute kidney injury
DKA: diabetic ketoacidosis
DM2: type 2 diabetes mellitus
DPP4is: dipeptidyl peptidase-4 inhibitors
eDKA: euglycemic diabetic ketoacidosis
eGFR: estimated glomerular filtration rate
FDA: US Food and Drug Administration
GDMT: guideline-directed medical therapy
HF: heart failure
SGLT2is: sodium-glucose cotransporter 2 inhibitors
